# Anticonvulsant effects of *antiaris toxicaria* aqueous extract: investigation using animal models of temporal lobe epilepsy

**DOI:** 10.1186/s13104-017-2488-x

**Published:** 2017-04-26

**Authors:** Priscilla Kolibea Mante, Donatus Wewura Adongo, Eric Woode

**Affiliations:** 1 Department of Pharmacology, Faculty of Pharmacy and Pharmaceutical Sciences, Kwame Nkrumah University of Science and Technology, Kumasi, Ghana; 2 University of Health and Allied Sciences, Ho, Ghana

**Keywords:** Hippocampus, Kainic acid, Pentylenetetrazole, Pilocarpine, Kindling

## Abstract

**Background:**

*Antiaris toxicaria* has previously shown anticonvulsant activity in acute animal models of epilepsy. The aqueous extract (AAE) was further investigated for activity in kindling with pentylenetetrazole and administration of pilocarpine and kainic acid which mimic temporal lobe epilepsy in various animal species.

**Results:**

ICR mice and Sprague–Dawley rats were pre-treated with AAE (200–800 mg kg^−1^) and convulsive episodes induced using pentylenetetrazole, pilocarpine and kainic acid. The potential of AAE to prevent or delay onset and alter duration of seizures were measured. In addition, damage to hippocampal cells was assessed in kainic acid-induced *status epilepticus* test. 800 mg kg^−1^ of the extract suppressed the kindled seizure significantly (*P* < 0.05) as did diazepam. AAE also produced significant effect (*P* < 0.01) on latency to first myoclonic jerks and on total duration of seizures. The latency to onset of wet dog shakes was increased significantly (*P* < 0.05) by AAE on kainic acid administration. Carbamazepine and Nifedipine (30 mg kg^−1^) also delayed the onset. Histopathological examination of brain sections showed no protective effect on hippocampal cells by AAE and nifedipine. Carbamazepine offered better preservation of hippocampal cells in the CA1, CA2 and CA3 regions.

**Conclusion:**

*Antiaris toxicaria* may be effective in controlling temporal lobe seizures in rodents.

**Electronic supplementary material:**

The online version of this article (doi:10.1186/s13104-017-2488-x) contains supplementary material, which is available to authorized users.

## Background

Epilepsy is a common neurological disorder which may be due to an imbalance between excitatory and inhibitory arms of the central nervous system—produced by a decrease in GABAergic and/or an increase in glutamatergic transmission [1].

Kindling and Status Epilepticus are the two most commonly used animal models of Temporal Lobe Epilepsy (TLE). Both models provide a dependable induction of a persistent, epileptic-like condition, despite their unique characteristics. Kindling is a simple phenomenon in which repeated induction of focal seizure discharge produces a progressive, highly reliable, increase in epileptic response to the inducing agent, usually electrical stimulation [2]. However, the use of chemical inducing agents, such as pentylenetetrazole has been shown to be equally effective [3, 4]. Acute administration of a high dose of pilocarpine in rodents is widely used to study the pathophysiology of seizures. It was first described by Turski et al. in 1983 [5, 6]. Pilocarpine-induced seizures reveal behavioural and electroencephalographic features that are similar to those of human temporal lobe epilepsy. Kainic acid, like pilocarpine, can also be used to induced a similar TLE or status epilepticus state in a variety of species using either systemic, intrahippocampal or intra-amygdaloid administrations [7].

Temporal lobe epilepsy is the most common form of complex partial seizures accounting for approximately 60% of all patients with epilepsy. Medial temporal lobe epilepsy which is the commonest temporal lobe epilepsy is also frequently resistant to medications and associated with hippocampal sclerosis. Management is challenging and often surgery has to be resorted to [8].

The plant *Antiaris toxicaria* (family Moraceae) is a common plant in Ghanaian forests. It has been employed traditionally as an analgesic and anticonvulsant [9]. Previous studies have shown that *Antiaris* possesses anticonvulsant activity in various acute murine models [10]. *Antiaris toxicaria* in this present study was evaluated to determine its properties in kindling models and post-status models of temporal lobe epilepsy. This investigation sought to determine if the extract possessed potential as an antiepileptogenic agent as well as efficacy in the management of temporal lobe epilepsy.

## Methods

### Plant material

Stem bark of *A. toxicaria* was harvested from the KNUST campus, Kumasi and identified by a staff member of the Pharmacognosy Department where a voucher specimen (KNUST/HM1/011/S007) has been retained in the herbarium.

### Preparation of *Antiaris toxicaria* aqueous extract

The dry stem bark was powdered using a commercial grinder. The coarse powder (431 g) was extracted by cold maceration with distilled water as solvent at room temperature for 5 days. The resultant filtrate was oven-dried to obtain 23.40%$${\raise0.7ex\hbox{${ w}$} \!\mathord{\left/ {\vphantom {{ w} w}}\right.\kern-0pt} \!\lower0.7ex\hbox{$w$}}$$ of *A. toxicaria* aqueous extract (AAE).

### Animals

Naïve male ICR mice (20–25 g) and Sprague–Dawley Rats (120-145 g) were obtained from Noguchi Memorial Institute for Medical Research, Accra, Ghana and kept in the departmental Animal House. Animals were maintained under laboratory conditions (room temperature; 12-h light–12-h dark cycle) in stainless steel cages (34 × 47 × 18 cm^3^) with wood shavings as bedding and allowed free access to water and food ad libitum. They were fed with normal commercial diet (GAFCO Ltd). Animals were tested in groups of eight. Groups were assigned randomly. Sample size was calculated using method of power analysis using the G-power software version 3.0.5. Experiments were carried out during the day. All animals were handled in accordance the Guide for the Care and Use of Laboratory Animals [11] and experiments were approved by the Faculty of Pharmacy and Pharmaceutical Sciences Ethics Committee, KNUST.

### Drugs and chemicals

Diazepam (DZP), pentylenetetrazole (PTZ) pilocarpine (PILO) and kainic acid (KA) were purchased from Sigma-Aldrich Inc., St. Louis, MO, USA.

### Kindling induction

PTZ kindling was initiated using a subconvulsive dose of PTZ 40 mg kg^−1^ body weight injected into the soft skin fold of the neck on every 2nd day (i.e. Day 1, Day 3, Day 5…). The PTZ injections were stopped when the control animals showed adequate kindling, i.e. Racine score of 5. After each PTZ injection, the convulsive behaviour of the rodent was observed for 30 min in an observation chamber. The resultant seizures were scored as follows: Stage 0 (no response); Stage 1 (hyperactivity, restlessness and vibrissae twitching); Stage 2 (head nodding, head clonus and myoclonic jerks); Stage 3 (unilateral or bilateral limb clonus); Stage 4 (forelimb clonic seizures); Stage 5 (generalized clonic seizures with loss of postural control). AAE was tested at doses of 200, 400 and 800 mg kg^−1^ body weight orally and diazepam (0.1, 0.3 and 1 mg kg^−1^, i.p). PTZ was injected 30 min after administration of test drugs. Control animals received 3 ml kg^−1^ of distilled water. Seven groups of eight animals each were used. Group 1 = distilled water-treated control group; groups 2–4 = AAE-treated groups and groups 5–7 = diazepam-treated group.

### Pilocarpine-induced Status epilepticus

Seizures were induced by an i.p. injection of pilocarpine (PILO) (300 mg kg^−1^, i.p.) into drug or vehicle-treated male rats. Rats were pre-treated with AAE (100–1000 mg kg^−1^, *p.o*.) or diazepam (0.3–3.0 mg kg^−1^, i.p.) for 30 or 15 min, respectively, before PILO injection. To reduce peripheral autonomic effects produced by PILO, the animals were pre-treated with *n*-*butyl*-bromide hyoscine (1 mg kg^−1^, i.p.) 30 min before PILO administration. Animals were placed in observation cages and observed via video recordings. Latency to and duration of seizures were scored.

### Rat kainate model

Animals were pre-treated with the plant extract 30 min as above before administration of kainic acid (10 mg kg^−1^, i.p.). Other animals were treated with carbamazepine (30 mg kg^−1^, *p.o*) and nifedipine (30 mg kg^−1^, *p.o*) 30 min before induction of convulsions. Animals were observed for wet dog shakes over a 1 h period [12]. Brains were harvested for histopathological examination after an hour. Tissues were fixed in 10% buffered formalin (pH 7.2). Dehydration was done with a series of ethanolic solutions, embedded in paraffin wax and processed for histological analysis. Coronal sections (2 µm thick) were cut and stained with haematoxylin-eosin for examination. The stained tissues were observed through an Olympus microscope (BX-51) and photographed by a chare-couple device (CCD) camera.

### Data analysis

Data were presented as mean ± S.E.M and significant differences between means determined by one-way analysis of variance (ANOVA) followed by Newman–Keuls’ post hoc test. Statistical analyses were carried out with Graph Pad Prism^®^ Version 5.0 (GraphPad Software, San Diego, CA, USA) and SigmaPlot^®^ Version 11.0 (Systat Software, Inc.). Data from 5 to 8 animals in each group were included in the analyses. *P* < 0.05 was considered significant in all cases. None were excluded.

## Results

### Effects in kindling

**Fig. 1:**
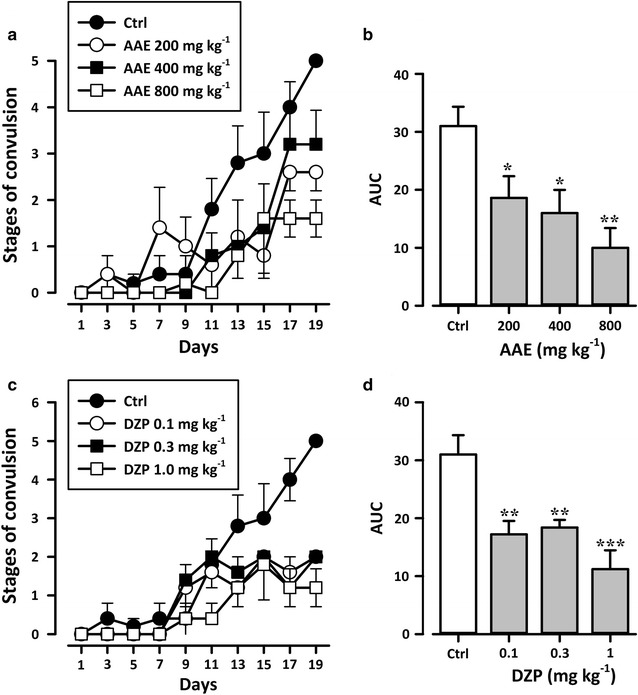
Effects of AAE (200, 400 and 800 mgkg^−1^, *p. o.*; **a** and **b**) and diazepam (0.1, 0.3 and 1 mgkg^−1^, i.p.; **c** and **d**) on the stages of convulsion attained in PTZ-induced kindling. Data are presented as group mean ± SEM (n = 8). *P < 0.05, ** P < 0.01, ***P < *0.001* compared with vehicle treated group (One-way analysis of variance followed by Newman–Keuls post hoc test)

**Fig. 2:**
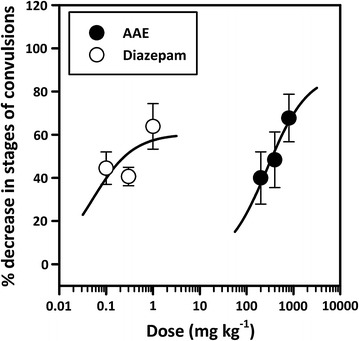
Dose-response curves of AAE and diazepam on the % decrease in stages of convulsions in PTZ-induced kindling. Each point represents mean ± S.E.M (n = 8)

In PTZ + vehicle-treated group, repeated administration of subconvulsive dose of PTZ (40 mg kg^−1^) on every alternate day for 20 days resulted in increasing convulsive activity leading to generalized clonic seizures (Racine score of 5). Administration of AAE in the dose of 200 and 400 mg kg^−1^ did not modify the course of kindling induced by PTZ significantly. However, a higher dose of 800 mg kg^−1^ suppressed the kindled seizure significantly (*P* < 0.05; Fig. 1a, b) as the group could not achieve a mean score of 5. The standard anticonvulsant diazepam significantly (*P* < 0.01; Fig. 1c, d) modified the course of kindling at all three dose levels compared to the control. ED_50_ obtained for the extract was 276.70 mg kg^−1^ compared to 0.05 mg kg^−1^ for diazepam. The extract was however more efficacious than diazepam achieving an E_max_ of 88.83% compared to 60.36% for diazepam (Fig. 2).

### Pilocarpine-induced status epilepticus

**Fig. 3:**
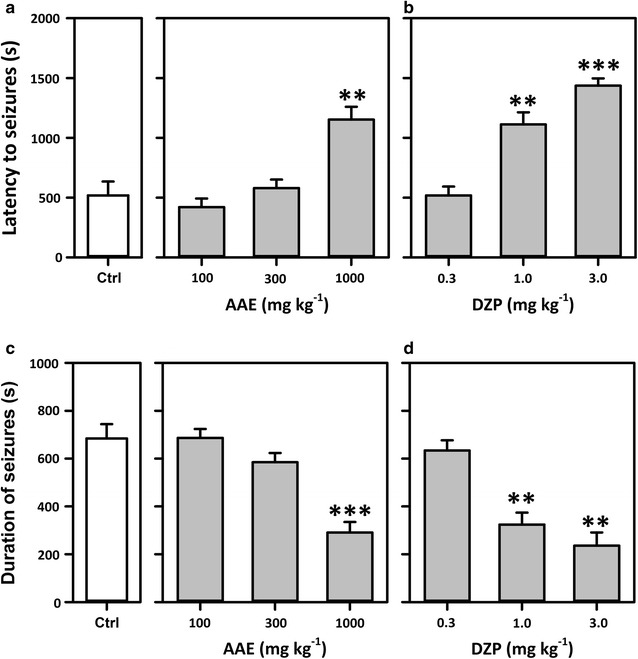
Effect of AAE (100–1000 mg kg^−1^, *p.o*.) and diazepam (0.3–3 mg kg^−1^, i.p.) on the latency to (**a** and** b**) and total duration of seizures (**c** and** d**) induced by PILO. Each* column* represents the mean ± SEM (n = 8). **P < 0.01, ***P < *0.001* compared to vehicle-treated group (One-way ANOVA followed by Newman–Keuls post hoc test)

**Fig. 4:**
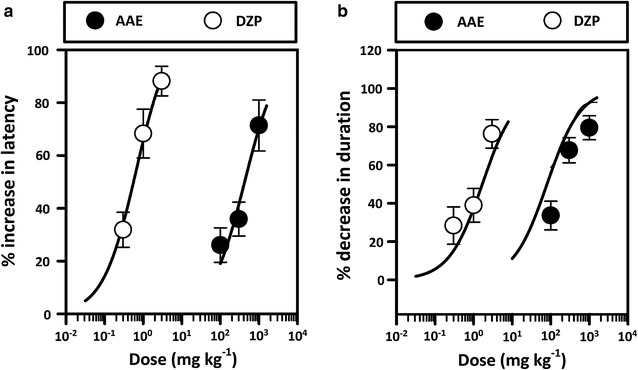
Dose-response curves of AAE and diazepam on the  % increase in latency (**a**) and  % decrease in durations (**b**) of status epilepticus induced with pilocarpine. Each point represents mean ± S.E.M (n = 8)

Pilocarpine induced behavioural changes including hypoactivity, tremor and myoclonic movements of the limbs progressing to recurrent myoclonic convulsions with rearing, falling, and status epilepticus. AAE produced significant effect (*P* < 0.01, Fig. 3a) on the latency to first myoclonic jerks as compared to control at the highest dose only. It had a similar effect on the total duration of seizures (Fig. 3c). Diazepam was used as the reference drug and it also significantly reduced the total duration of seizures (P < 0.01, Fig. 3d) and latency (*P* < *0.001*, Fig. 3b) at 1 and 3 mg kg^−1^. Diazepam was more potent than the extract in increasing the  % latency with an ED_50_ of 0.66 mg kg^−1^ as against 424.50 mg kg^−1^ for the extract (Fig. 4a). Diazepam was also more efficacious achieving an E_max_ of 108.90% compared to 100% for the extract. Likewise, for the % duration AAE produced ED_50_ = 80.06 mg kg^−1^ and E_max_ = 100% while the standard diazepam achieved ED_50_ = 1.67 mg kg^−1^ and E_max_ = 100% (Fig. 4b).

### Effects in rat kainate model

**Fig. 5:**
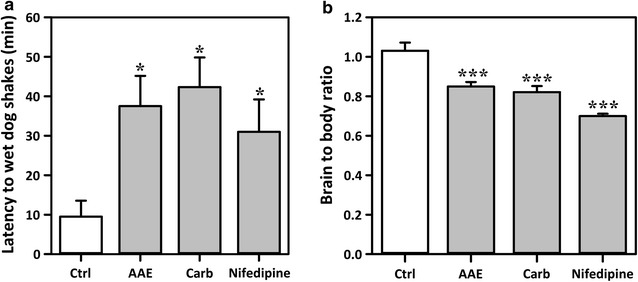
Effects of AAE (400 mgkg^−1^, *p.o.*), carbamazepine (30 mgkg^−1^, *p.o.*) and nifedipine (30 mgkg^−1^, *p.o*.) on the latency to wet dog shakes (**a**) and  % brain to body ratio (**b**) in rat kainate model. Data are presented as group mean ± SEM (n = 8). *P < 0.05, ***P < *0.001* compared to vehicle-treated group (One-way analysis of variance followed by Newman–Keuls’ post hoc Test)

**Fig. 6:**
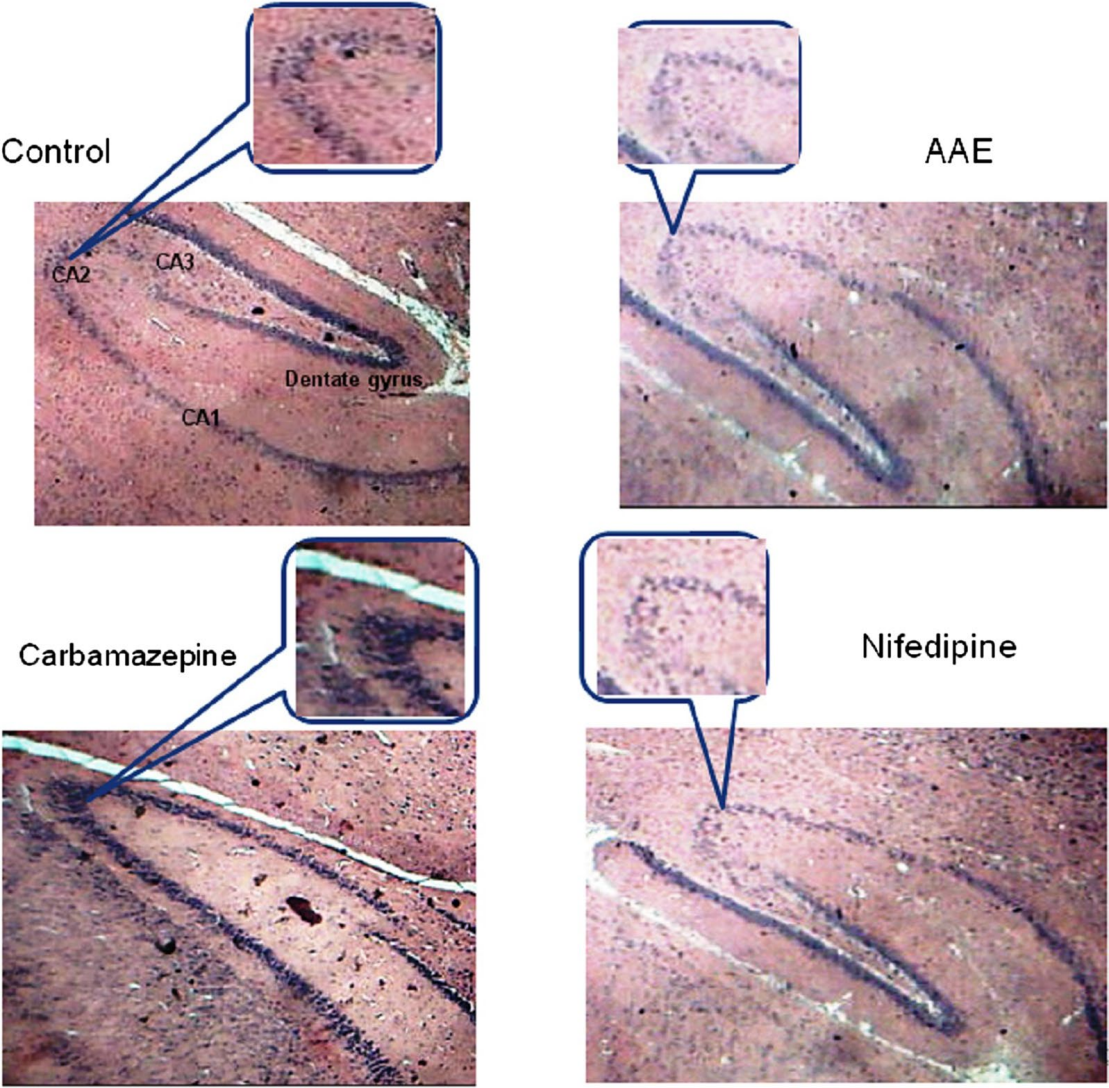
Photomicrographs of coronal sections of the brain of rats AAE (400 mg kg^−1^), carbamazepine (30 mg kg^−1^) and nifedipine (30 mg kg^−1^) on kainate– induced hippocampal damage (H & E, ×100)

Kainic acid (10 mg kg^−1^, i. p) produced wet dog shakes in all animals. AAE (400 mg kg^−1^) produced a significant (*P* < 0.05) increase in time taken to the onset of wet dog shakes (Fig. 5a). Carbamazepine (30 mg kg^−1^) and Nifedipine (30 mg kg^−1^) also delayed the onset. Histopathological examination of the coronal section of the brain showed no protective effect on hippocampal cells by AAE and nifedipine. Carbamazepine offered better preservation of hippocampal cells in the CA1, CA2 and CA3 regions (Fig. 6). The brain to body ratio decreased significantly (*P* < 0.001; Fig. 5b) with all three treatments.

## Discussion

Kindling is a chronic model of epilepsy and epileptogenesis. Repeated administration of a subconvulsive dose of PTZ (a blocker of the GABA_A_ receptor) results in the progressive intensification of convulsant activity, culminating in a generalized seizure [3, 4]. The highest dose of AAE (800 mg kg^−1^) significantly delayed progression of convulsion similarly to diazepam.

Many substances interacting with GABA receptors have been shown to produce potent anticonvulsant effects on seizures in previously kindled animals [2, 13]. It has been shown that AAE produces anticonvulsant effects by interacting with the GABA_A_ receptor. The fact that it acts via GABAergic mechanisms may be a possible explanation for anticonvulsant effects being exhibited in the kindling model.

There is some evidence that free radicals are actively involved in physiological processes during oxidative stress induced by administration of convulsants [14]. Of all the free oxygen radicals that occur in vivo, the hydroxyl-free radicals (OH^−^) are considered to be most hazardous [15–17]. Different mechanisms may lead to the increase of the free radicals in PTZ-induced convulsions. It may be assumed that further reason exist for the increased formation of OH^−^ in kindled animals during PTZ seizure, such as reduced activity of superoxide dismutase (SOD), a major defence system for counteracting the toxic effects of reactive oxygen species such as O^2−^. However, antioxidant activity of AAE has not been firmly established.

AAE exhibited anticonvulsant effects against pilocarpine-induced seizures. Pilocarpine is a cholinergic agonist, widely used experimentally to induce limbic seizures in structures containing a high concentration of muscarinic receptors such as the cerebrum [18–20]. Status Epilepticus produces significant decreases in M _1_, M _2_, and GABAergic receptor densities [21] and hence neurotransmission. Freitas et al. have also reported in 2004 on increased levels of superoxide dismutase and catalase and reductions in acetylcholinesterase enzymatic activities in the rat frontal cortex and hippocampus. During pilocarpine-induced seizures and SE in adult rats, lipid peroxidation processes are increased [21, 22] suggesting free radical involvement in the pilocarpine-induced brain damage. Certain antioxidants, such as ascorbic acid, have therefore been shown to possess anticonvulsant activity against pilocarpine-induced SE [22, 23]. Muscarinic receptor stimulation is alleged to be responsible for the onset of pilocarpine-induced seizures, while glutamate acting on NMDA receptors sustains seizure activity [18]. Analysis of the brain morphology after pilocarpine administration demonstrates that the CA1 hippocampal neurones and the hilus of dentate gyrus are predominantly susceptible to neuronal cell loss [5]. Neuronal cell death during SE occurs largely by excitotoxic injury caused by the activation of glutamatergic pathways [6, 24]. Thus, the ability of AAE to attenuate seizures induced by pilocarpine could be attributed to cholinergic antagonism at the M_1_ or M_2_ receptors, increase in GABA, and/or its receptor densities, decrease in glutamate levels or through antioxidant pathways. Activation of potassium ion conductance can also contribute as it results in inhibition of the release of glutamate [25, 26]. AAE may therefore have potential in the management of *status epilepticus*.

Kainic acid is a neuro excitotoxic analogue of glutamate used in studies of epilepsy to model experimentally induced limbic seizures [27, 28]. Kainate-treated rats may respond differently. Some may produce wet dog shakes (equivalent to a class III seizure on the Racine scale) or more severe seizures [29]. Previous studies have shown pattern of neurodegeneration in the hippocampus with high concentration of high affinity KA binding sites (CA3 pyramidal cells of the hippocampus) [7, 30]. The dentate gyrus from kainate-treated rats has shown the presence of mossy fibre sprouting in the inner molecular layer [7, 31]. Examination of the hippocampus after seizures revealed hippocampal damage, especially in the CA3 and CA2 regions as shown in the photomicrographs. The extract showed no significant protection against such damage even though it significantly delayed the latency to wet dog shakes. This implies that the extract possesses general anticonvulsant properties but offers no protection against morphological changes. The kainate-treated rat model is used to study temporal lobe epilepsy. However, similarity of seizure occurrence to human temporal lobe epilepsy has not been studied comprehensively. But there are several characteristics of the seizures that resemble temporal lobe epilepsy in humans. For instance, some of the animals produce a few observed motor seizures (even with several months of observation) after a latent period, while other animals have seizures at a frequency as high as 1–2 hz which is reminiscent of epilepsy in the human population [8]. The rats often demonstrate confusion (e.g. hyperactive exploration of their cage) after a seizure, resembling the post-ictal confusion in most humans with temporal lobe epilepsy [32, 33]. Rats treated with the extract exhibited fewer seizures as compared to the control implying a possibility that it might be effective in the treatment of temporal lobe epilepsy. The CA1 and CA3 regions of the hippocampus are also known to possess one of the highest densities of the dihydropyridine receptors in the rat brain [34, 35]. The results of many experimental studies have shown that calcium channel blockers are effective against several different types of seizures [35–37]. Hence, nifedipine proved its effect in this model. As much as these experiments model human temporal lobe epilepsy, caution has to be exercised in translating the results directly to man without the needed clinical trials. The results however further lend scientific credence to the traditional use of *A. toxicaria* as an antiepileptic.

## Conclusion

*Antiaris toxicaria* possesses anticonvulsant properties in kindling and status epilepticus murine models and may be antiepileptogenic and a candidate for the management of temporal lobe epilepsy (Additional file 1).

## Additional file

**Additional file 1.** Raw data.
